# Evaluation of ITGB1 expression as a predictor of the therapeutic effects of immune checkpoint inhibitors in gastric cancer

**DOI:** 10.1186/s12876-023-02930-0

**Published:** 2023-09-04

**Authors:** Chao Xu, Xiao-Li Xie, Ning Kang, Hui-Qing Jiang

**Affiliations:** 1 Department of Gastroenterology, The Second Hospital of Hebei Medical University, Hebei Key Laboratory of Gastroenterology, Hebei Institute of Gastroenterology, Hebei Clinical Research Center for Digestive Diseases, No. 215, Heping West Road, Shijiazhuang, 050000 Hebei Province China; 2 Handan Central Hospital, No.15, Zhonghua Road, Handan, 056001 Hebei Province China

**Keywords:** ITGB1, Gastric cancer, Bio-informatics, Predictive value, Immunohistochemistry, Immunotherapy

## Abstract

**Background:**

Gastric cancer (CC) is a disease with high incidence and mortality rate. Immunotherapy is an important method for gastric cancer while lack of effective predictor. Integrins play an important role in the development. We aimed to explore the predictive value of β1 integrin (ITGB1) as a predictor of immunnotherapy in gastric cancer.

**Methods:**

Differential expression analysis was conducted using the Gene Expression Profiling Interactive Analysis (GEPIA) 2.0 and GEO databases. GEPIA data were used to evaluate the prognostic value of ITGB1 in gastric cancer (GC). Transcriptomic and clinical data of GC and normal tissues were downloaded from The Cancer Genome Atlas database, and the TIMER database was used to evaluate the association between ITGB1 and immune infiltration. Time-dependent receiver operating characteristic (ROC) curve analysis was used to determine the prognostic value of ITGB1. To verify ITGB1 expression at the protein level, immunohistochemical staining was conducted. In addition, to analyze the correlation of ITGB1 with PD-1 and PD-L1, we examined levels of PD-1 and PD-L1 by IHC and determined the predictive value of ITGB1 for anti-PD-1 therapy in GC by ROC curve analysis.

**Results:**

Compared with normal tissues, analysis of GEPIA and data at protein levels showed significantly higher expression of ITGB1 in GC. In addition, higher expression of ITGB1 was associated with worse pathological G-staging and tumor T-staging, which suggested that ITGB1 is a risk factor for poor prognosis in GC. The level of ITGB1 expression was positively correlated with CD8 + T cells, neutrophils, macrophages, and dendritic cells. ITGB1 expression was also correlated with PD-L1 expression, and this was further verified at the protein level by immunohistochemical analysis. The area under the ROC curve was 0.808.

**Conclusion:**

ITGB1 may be a promising prognostic biomarker and effective predictor for anti-PD-1 therapy in GC.

**Trial registration:**

Retrospectively registered.

**Supplementary Information:**

The online version contains supplementary material available at 10.1186/s12876-023-02930-0.

## Introduction

Gastric cancer (GC) is one of the most common malignant diseases and the fourth leading cause of cancer-related deaths worldwide [1]. A large majority of tumors are detected at an advanced stage, which means that the opportunity for surgery may be lost. Despite the use of fluoropyrimidine combined with platinum as first-line therapy, and taxane agents as second-line therapy for advanced GC, with trastuzumab for HER2-positive cases, the prognosis of the disease remains unsatisfactory, with median survival less than 1 year [2–5]. Recently, treatment with immune checkpoint inhibitors (ICIs) has become a promising choice in several malignancies [6–10]. PD-1, a 288-amino acid protein, is a negative costimulatory receptor that belongs to the CD28 family of immunoglobulins and expresses on the surface of activated T cells. When PD-1 binds to its receptor on tumor or immune cells, immune responses are suppressed. This can inhibit the function of cytotoxic T cells, which lead to immune escape [11]. Based on several clinical trials, ICIs are now approved for second-/third-line or late-line therapy for advanced GC [12]. Although ICIs have been considered as a breakthrough in cancer treatment, low response rate and therapeutic resistance remain significant challenges. In total, the average response rate is 20%–30%, while the lack of effective biomarkers is one of the most urgent issues [13].

The integrity of epithelium is maintained by various inter-cellular connections and cell polarity is also important. Epithelial-mesenchymal transition (EMT), the process by which epithelial cells obtain a mesenchymal phenotype, plays an important role in tumor immunosuppression and immune evasion. For example, EMT-induced immune escape causes cancer progression [14]. GC cells are stimulated by different signaling molecules such as growth factors, hypoxia, inflammatory factors, and metabolites in the tumor micro-environment, while stress also activates related signaling pathways. These signals stimulate downstream transcription factors to participate in the EMT process, thus affecting cell proliferation, migration, invasion, and other biological process. At present, various signaling pathways (including the WNT/β-catenin [15], TGF-β/SMAD [16], MAPK, Notch [17, 18], and PI3K/AKT pathways [19]) are known to promote the development of EMT and ultimately stimulate migration and invasion of GC cells. Previous studies have revealed that [20], EMT is associated with the activation of several immune checkpoint molecules, including PD-L1. Accordingly, by identifying genes related to both EMT and immune infiltration, we hope to find effective genes for therapeutic responses prediction to checkpoint inhibitors.

In this study, to find markers capable of predicting responses to immunotherapy, we searched public data sets and selected genes related to EMT and immune infiltration. After removing known genes, we found that β1 integrin (ITGB1) appears to be an effective biomarker for GC immunotherapy. As the main receptor of fibronectin (FN), ITGB1 is one of the most important cell adhesion molecules. When FN combines with its receptor, increased expression of ITGB1 induces a decrease in FN levels, degradation of extracellular matrix (ECM), acceleration of EMT, and finally culminating in cancer or metastasis. Studies have confirmed that [21], high levels of ITGB1 are found during the development of various tumors and are also associated with immune cell infiltration. In addition, researchers found that [22] ropivacaine regulates the function of colon cancer cells by targeting the expression of the ITGB1 protein and affecting the activation of its downstream signaling pathways. The study of Zhuang et al. demonstrated that [23], ITGB1 overexpression was significantly associated with advanced American Joint Committee on Cancer (AJCC) stage and histologic grade as well as worse prognosis in pancreatic cancer. Moreover, ITGB1 enhanced the radiotherapy resistance of human non-small cell lung cancer [24], while tumor progression was particularly noted in ITGB1-positive GC [25].

Despite these findings, the role of ITGB1 expression in GC remains unclear. Therefore, ITGB1 expression in GC and its effect on immune cell infiltration as well as its influence on patient prognosis were discussed in this study. Correlations of ITGB1 with immune infiltration and immune checkpoints were also determined, while gene expression of immune checkpoint was verified by analysis of pathological sections. Thus, this study provides a foundation for further research into immune checkpoint regulation and corresponding therapy.

## Methods

### Identifying the EMT-immune-related differential expression genes

Datasets of GSE118916 and GSE79973 in the GEO database (https://www.ncbi.nlm.nih.gov/geo/) contain 15 and 10 gastric cancer and matched normal tissues respectively. Differentially expressed genes (DEGs) between normal and GC samples were calculated by GEO2R, with |logFC|> 1 and adjust *P* value < 0.05 were the screening criteria. InnateDB (https://www.innatedb.com/) database and Immport (https://immport.org/shared/home) database were used for detection of DEGs related with immune infiltration. EMT-related genes were defined by EMTome (www.emtome.org). DEGs between normal (GTEx) and GC (TCGA-COAD) were calculated by GEPIA 2.0 (http://gepia2.cancer-pku.cn/index.html). K-M survival curves were plotted. Moreover, Pubmed literature searching was conducted to identify the target genes with research meaning and fewer studies in gastric cancer.

### Expression validation of ITGB1 in gastric cancer

The dataset GSE79973 in the GEO database (https://www.ncbi.nlm.nih.gov/geo/) contain 10 cases of GC and matched normal tissues. Expression data for gene name transformation were downloaded on the GPL570 chip platform and utilized to analyze the expression of IGTB1 in GC and matched normal tissues. Normal (GTEx) and GC (The Cancer Genome Atlas stomach adenocarcinoma [TGCA-STAD]) samples were analyzed using the GEPIA 2.0 online tool (http://gepia2.cancer-pku.cn/index.html) to further validate ITGB1 expression.

### Prognostic value and clinicopathological features of ITGB1 in gastric cancer

RNA-seq RPKM data and clinical data of gastric adenocarcinoma were downloaded from the TCGA database (https://portal.gdc.cancer.gov/). In total, 375 GC samples were included in the following analyses. By using the GEPIA, correlation of ITGB1 expression with pathological grade and clinical stage (T, N, and M stages) were analyzed, then prognostic value of ITGB1 in GC is determined through receiver operating characteristic (ROC) curves. Kaplan–Meier plotter (https://kmplot.com/analysis/) were used to confirm the results of the prognostic value of ITGB1.

### Analysis of immune infiltration and correlations of immune checkpoints with ITGB1

R language was used to analyze immune infiltration status in TCGA gastric cancer data by CIBERSORT. In addition, the “Gene” module of TIMER 2.0 (http://timer.comp-genomics.org/) was used to evaluate the correlation of ITGB1 expression and immune infiltration. Then, we utilized the “Gene_corr” module to calculate the correlation coefficients of ITGB1 and classical immune cell markers by Spearman. Correlation of ITGB1 and immune checkpoints were also analyzed using the same method.

### Co-expression and pathway enrichment analysis of ITGB1

By using STRING database (https://string-db.org/), we regulated the “max number of interactors to show” to “1st shell no more than 50” in order to carry out the co-expressional analysis of the hub genes. Interactions of the genes were displayed by GeneMANIA (http:// www.genemania.org). Moreover, we conducted the pathway enrichment analysis of the genes by the Metascape database (http://metascape.org) and showed the top 20 items.

### Expression validation of ITGB1 by immunohistochemistry

To verify the expression of ITGB1 at the protein level, we obtained 27 paraffin-embedded tissues of GC from the pathology department. All patients in our study were primary GC cases with no prior treatment. For immunohistochemical (IHC) staining, we used anti-PD-1 mouse monoclonal antibody, integrin beta 1 rabbit polyclonal Ab (from Chengdu Zhengneng Biotechnology Co., Ltd.), and anti-PD-L1 monoclonal antibody (from Wuhan Sanying Biotechnology Co., Ltd.). All sections were deparaffinized in xylene and rehydrated in ethanol. Next, we used sodium citrate antigen retrieval solution (Solarbio) for heat-mediated antigen retrieval. After treating with 3% H_2_O_2_ (30 min) and blocking in normal goat serum (1 h), the tissue sections were incubated with ITGB1 (concentration 1:50), PD-1 (concentration 1:200), and PD-L1 (concentration 1:200) antibodies overnight (4℃). Then, we stained the tissue sections with secondary antibody (OriGene SP-9001) for 20 min at room temperature, followed by horseradish-labeled streptavidin (OriGene SP-9001) for 15 min. Diaminobenzidine (OriGene ZLI-9018) was used as a substrate for the peroxidase reaction, and the sections were then stained with hematoxylin (Leagene). ITGB1 was mainly expressed on the cell membrane and in cytoplasm, while PD-1 and PD-L1 were detected on the cell membrane. For all tissues, integrated optical density was calculated using Image-Pro Plus 6.0 software. Subsequently, correlations of ITGB1 and PD-1 or PD-L1 were determined and ROC curves for predicting the therapeutic effect of immunotherapy were mapped using SPSS 22.0. The involvement of human participants in the study was approved by the Ethics Committee of the Handan Central Hospital. Patients provided their written informed consent to participate in this study.

### Expression correlation of ITGB1 and PD-L1 in gastric cancer by in vitro cell experiments

In order to further verify the expression correlation of ITGB1 and PD-L1 in gastric cancer, we carried out in vitro cell experiments including cell culture and transfection, western blotting. The gastric carcinoma cell line HGC-27 was cultured in Mccoy’s 5A medium containing 10% fetal bovine serum (FBS) in a humidified incubator with 5% CO_2_ at 37 °C. RNA oligo (siRNA-ITGB1/siRNA-control) (from Suzhou Jima Biotechnology Co., Ltd.) was used for knockdown experiment. Proteins from cell pelleta were extracted using RIPA lysate (RIPA: PMSF = 100: 1). Cell lysates were eliminated by centrifugation at 4 °C for 10 min at 8,000 rpm. Five microgram aliquots of protein was separated electrophoretically on 12.5% SDS-PAGE gels and transferred to a nitrocellulose membrane. Nonspecific binding sites were blocked using a buffer with 5% nonfat dry milk in TBST for 1 h at 37 °C. The membranes were then incubated with appropriate antibodies overnight at 4 °C, washed 3 times with TBST, and incubated with horseradish peroxidase-conjugated anti-rabbit secondary antibody, for 1 h at 37 °C. According to the manufacturer’s protocol, bands were visualized and scanned with enhanced chemiluminescence regents. The following antibodies were used for western blot analysis: anti-PD-L1 monoclonal antibody (Proteintech Co., USA), integrin beta 1 polyclonal Ab (Proteintech Co., USA).

## Results

### Screening of EMT-immune-related differential expression genes

**Fig. 1:**
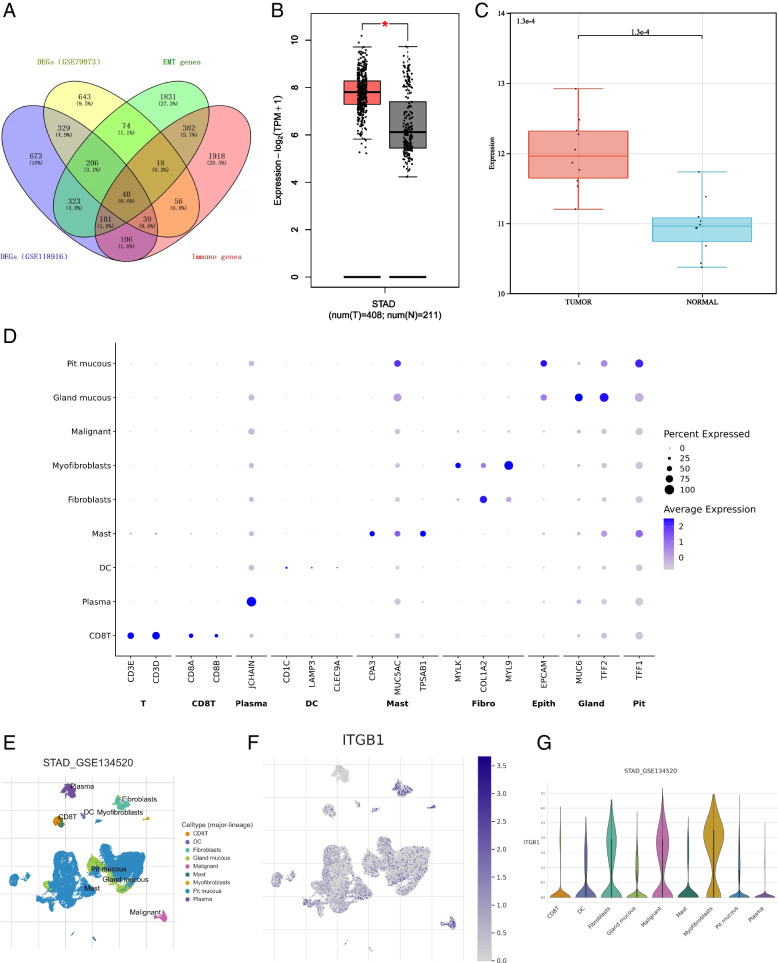
Relation of ITGB1 expression and prognosis. **A** Venn diagram for selecting DEG. **B** Higher expression of ITGB1 in GC from GEPIA 2.0 database, *P < 0.05. **C** Higher expression of ITGB1 in tumors from GSE79973 dataset. **D**-**E** Corresponding markers to do cluster defining and different cell types. **F**-**G** Higher density of ITGB1 in myofibroblasts and malignant cell

EMT and tumor immunity are considered to be important drivers of tumor progression, and the literature shows that EMT is also associated with the activation of some ICIs [26]. Genes involved in both biological process maybe important. 1817 differential expression genes were selected by the GSE118916 dataset, and 1406 differential expression genes were selected by the GSE79973 dataset. We obtained 2660 immune-related genes from InnateDB and Immport database, while 2975 EMT-related genes from EMTome database. Next, we obtained 40 EMT-immune-related DEGs after intersection of DEGs from GSE118916 dataset and GSE79973 dataset. Then, single gene prognostic analysis using GEPIA2.0 and literature search of Pubmed were undertook. Finally, ITGB1 was found to have important prognostic value in GC and was defined as the target gene for further research (Fig. 1A).

### ITGB1 is up-regulated in gastric cancer

Analysis of GTEx and TCGA data using GEPIA 2.0 confirmed that, comparing with normal tissues, ITGB1 levels were significantly up-regulated in GC tissues (*, *P* < 0.05) (Fig. 1B). Analysis of RNA-seq dataset GSE79973 of GC showed that ITGB1 expression levels were up-regulated in GC samples; a boxplot of the results indicated significantly higher levels of ITGB1 in tumors (Fig. 1C). The results above suggested that up-regulation of ITGB1 in GC may be a carcinogenic factor.

### Expression density of ITGB1 is higher in malignant cells

Furthermore, as the traditional bulk profiles represent the average expression levels of the constituent cells, it could not reflect the specific expression of different cell types. So, we evaluate the expression of ITGB1 at the single-cell level. We used the corresponding markers to do cluster defining and showed the different cell types in Fig. 1D, E. The expression density of ITGB1 was relatively higher in myofibroblasts and malignant cells, which could confirm the results above (Fig. 1F, G).

### ITGB1 is correlated with poor prognosis and tumor progression

**Fig. 2:**
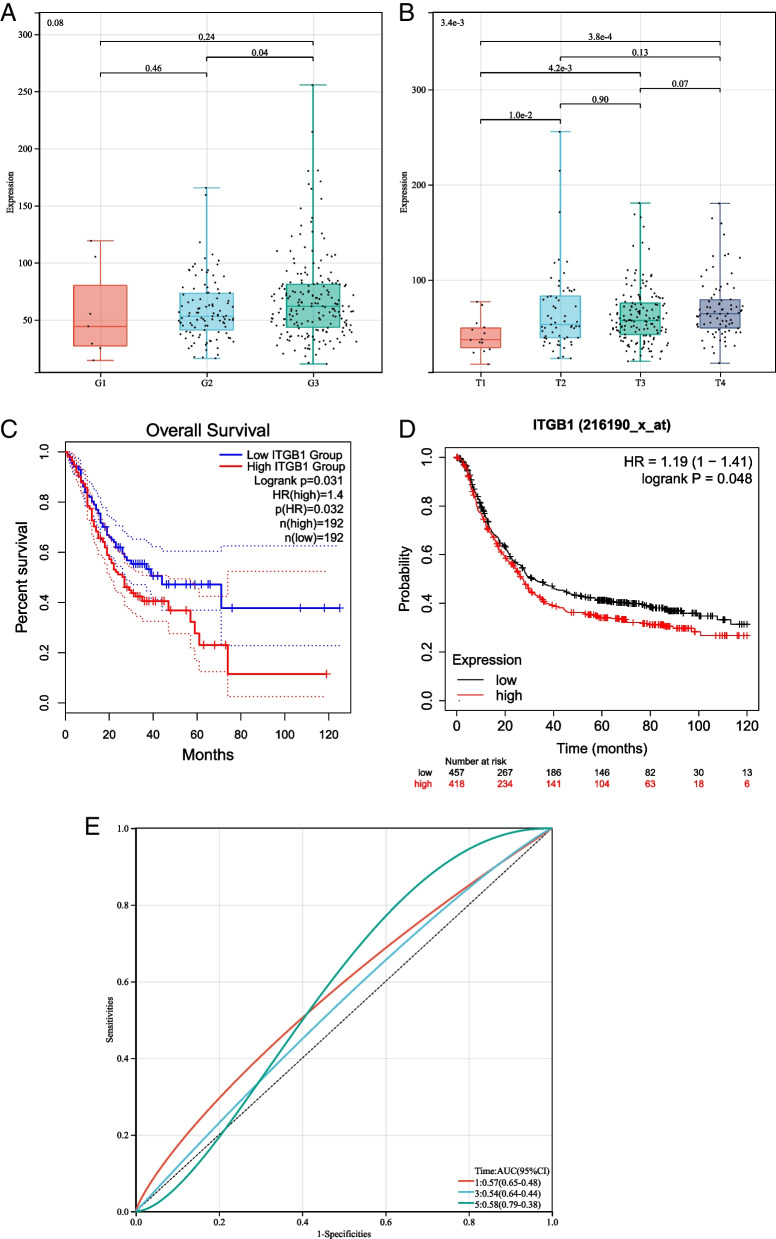
**A** Positive correlation between pathology G Degree and ITGB1 expression quantity. **B** Positive correlation between tumor T staging and ITGB1 expression quantity. **C** Lower overall survival rate of patient with higher expression of ITGB1 from TCGA. **D** Lower overall survival rate of patient with higher expression of ITGB1 from GEO. **E** Time dependence ROC indicate ITGB1 as a promising biomarker for prognostic prediction

To further analyze the prognostic value of ITGB1, we conducted correlation analysis with some clinical indicators using the TCGA dataset. The results showed that ITGB1 expression was positively associated with pathology G-stage and tumor T-stage, which indicating that ITGB1 may promote GC progression (Fig. 2A, B). Furthermore, to analyze the effect of ITGB1 on overall survival in GC, a Kaplan–Meier plot was constructed using the GEPIA 2.0 database. The Kaplan–Meier plot showed that high levels of ITGB1 expression resulted in relatively low overall survival (OS) (Fig. 2C). Similarly, the results of the Kaplan–Meier plotter verified that ITGB1 was a promising biomarker in gastric cancer (Fig. 2D). This result also indicated that high levels of ITGB1 resulted in poor prognosis. Time-dependent ROC analysis showed that the area under the curve for ITGB1 was relatively low. Accordingly, based on the results above, ITGB1 may be a promising biomarker for prognosis in GC (Fig. 2E).

### ITGB1 is correlated with immune infiltration

**Fig. 3:**
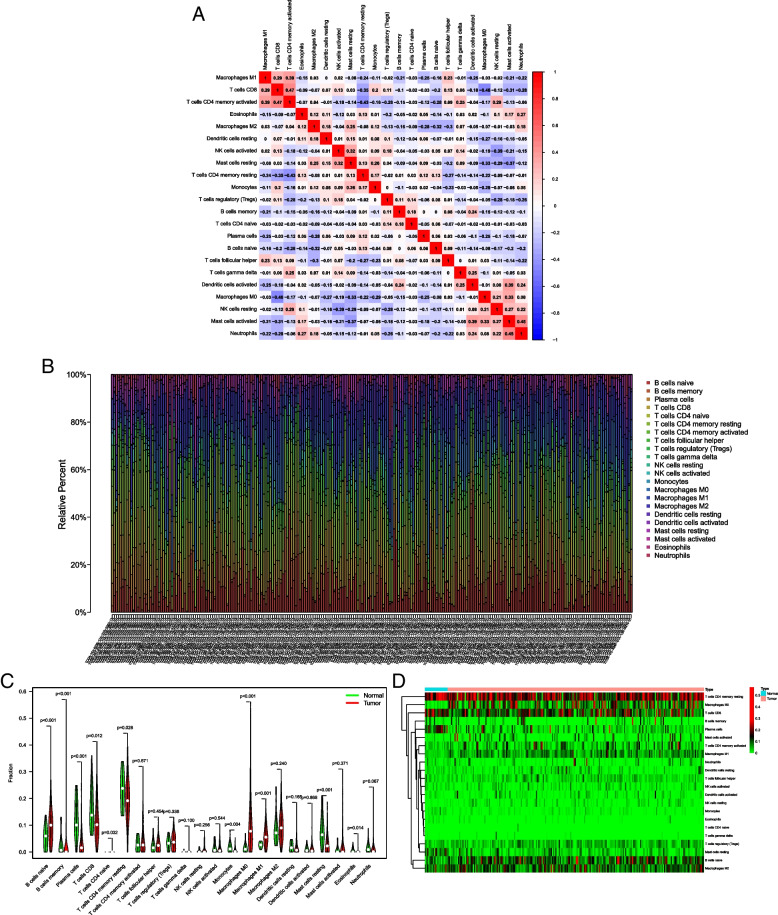
Immune related analysis in gastric cancer. **A** Immune infiltration of each TCGA-STAD sample. **B** Correlation among different immune cell types in GC. **C** The violin plot of immune infiltration between gastric cancer and normal samples. **D** The heat plot of immune infiltration between gastric cancer and normal samples

**Fig. 4:**
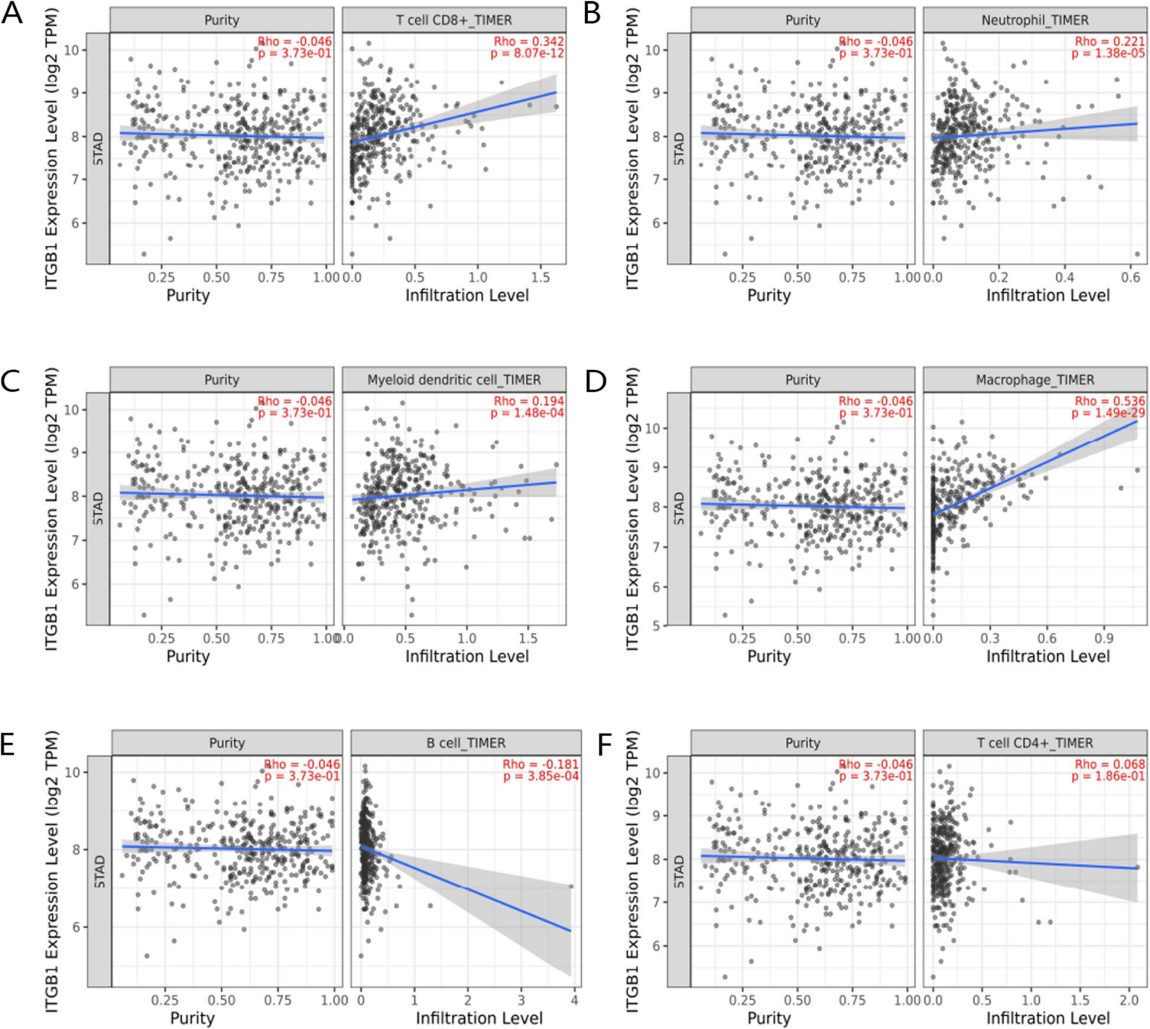
Correlation of ITGB1 expression with immune cells. **A**-**D** Positive correlation between ITGB1 expression levels and infiltration of CD8 + T cells, Neutrophil, dendritic cells and macrophages. **E** Negative correlation between ITGB1 expression levels and infiltration of B cells. **F** No significant correlation between ITGB1 expression levels and infiltration of CD4 + T cells

Next, we analyzed the influence of ITGB1 on the immune microenvironment. Immune infiltration for each TCGA-STAD sample and the correlations of immune cells with different types are shown in Fig. 3A, B. Differential immune infiltration in GC and normal samples are displayed in Fig. 3C, D. Subsequently, to explore the immune regulatory mechanism of ITGB1, the TIMER 2.0 database was used for correlation analysis of immune infiltration and immune markers. ITGB1 expression was positively correlated with infiltration of CD8 + T cells, neutrophils, dendritic cells and macrophages, while negatively correlated with B cells and no correlation with CD4 + T cells (Fig. 4A-F). Meanwhile, ITGB1 expression was positively with immune markers (Table 1). These findings were consistent with the TCGA-STAD results, which illustrated a strong correlation between ITGB1 and immune infiltration.

**Table 1:** Correlation of ITGB1 and Immune Cells

**Cell Type**	**Gene Markers**	**COR**	***P *value**
CD8+T cell	CD8A	0.119	2.09e-2*
	CD8B	0.066	2.03e-1
Th1 cells	TBX21	0.075	1.45e-1
Th2 cells	GATA3	0.202	7.32e-5*
Treg	FOXP3	0.069	1.81e-1
	CCR8	0.263	2e-7*
B cells	FCRL2	0.039	4.5e-1
	MS4A1	0.027	5.96e-1
	CD70	0.02	7.01e-1
Neutrophils	FPR1	0.279	3.23e-8*
	CSF3R	0.162	1.59e-03*
	SIGLEC5	0.297	3.76e-9*
Marcrophages	CD68	0.163	1.42e-03*
	CD84	0.292	6.93e-9*
	CD163	0.417	2.27e-17*

*P<0.05

### ITGB1 is positively correlated with immune checkpoints, especially PD-L1 and TIM

To further analyze the immune regulatory mechanism of ITGB1, we investigated potential correlations of ITGB1 expression and immune checkpoints. The results demonstrated that ITGB1 was positively correlated with the expression of TIGIT, CTLA4, PD-L1, and TIM. The highest correlation coefficients were obtained for PD-L1 and TIM (> 0.2) (Table 2). Thus, rather than decreasing immune infiltration, ITGB1 may up-regulate immune checkpoints to influence immune function and ultimately promote the progression of GC.

**Table 2:** Correlation between ITGB1 and Immune Checkpoints

	**COR**	***P *value**
TIGIT	0.127	1.34e-2*
CTLA4	0.103	4.45e-2*
CD274 (PD-L1)	0.221	1.4e-5*
PDCD1 (PD-1)	0.007	8.85-1
LAG3	0.042	4.18e-1
HAVCR2 (TIM)	0.29	8.97e-9*

*P<0.05

### ITGB1 regulates the development of gastric cancer through various signaling pathways

**Fig. 5:**
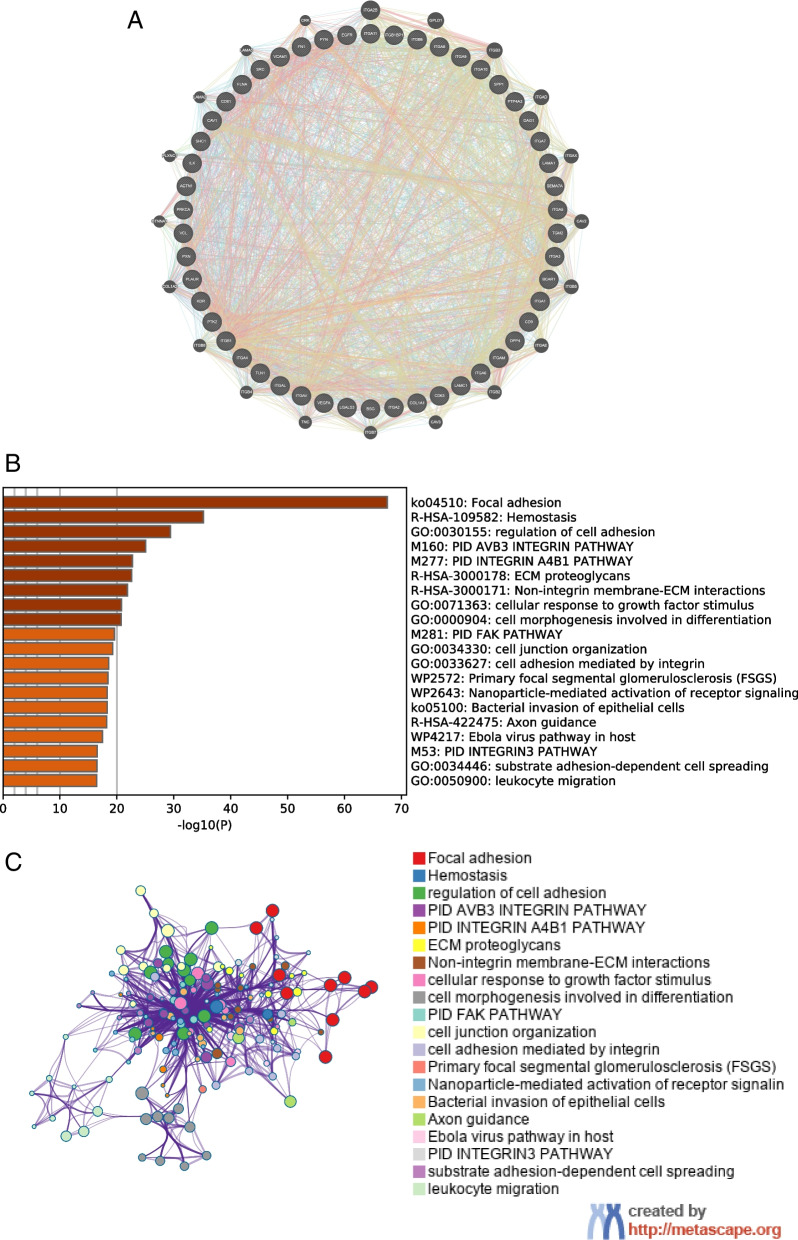
Co-expression and pathway enrichment analysis of hub genes. **A** Display of co-expressional network by GeneMANIA. **B** Pathway enrichment analysis of hub genes by Metascape. **C** Interactions among the enriched pathways

To further understand the mechanism of ITGB1 in gastric cancer, co-expression and pathway enrichment analyses were carried out. 50 co-expressed genes of ITGB1 were obtained by the STRING database, and co-expression interactions was displayed by GeneMANIA (Fig. 5A). Then pathway enrichment analysis of all the co-expressed genes was done using Metscape. Figure 5B, C showed that, ITGB1 was enriched in pathways related to cell adhesion, tumor metastasis, cellular response to growth factor stimulus, leukocyte migration and so on. The above results indicate that ITGB1 may regulate the prognosis of gastric cancer through various signaling pathways.

### Validation of ITGB1 expression and correlation with PD-1 and PD-L1 by immunohistochemistry

**Fig. 6:**
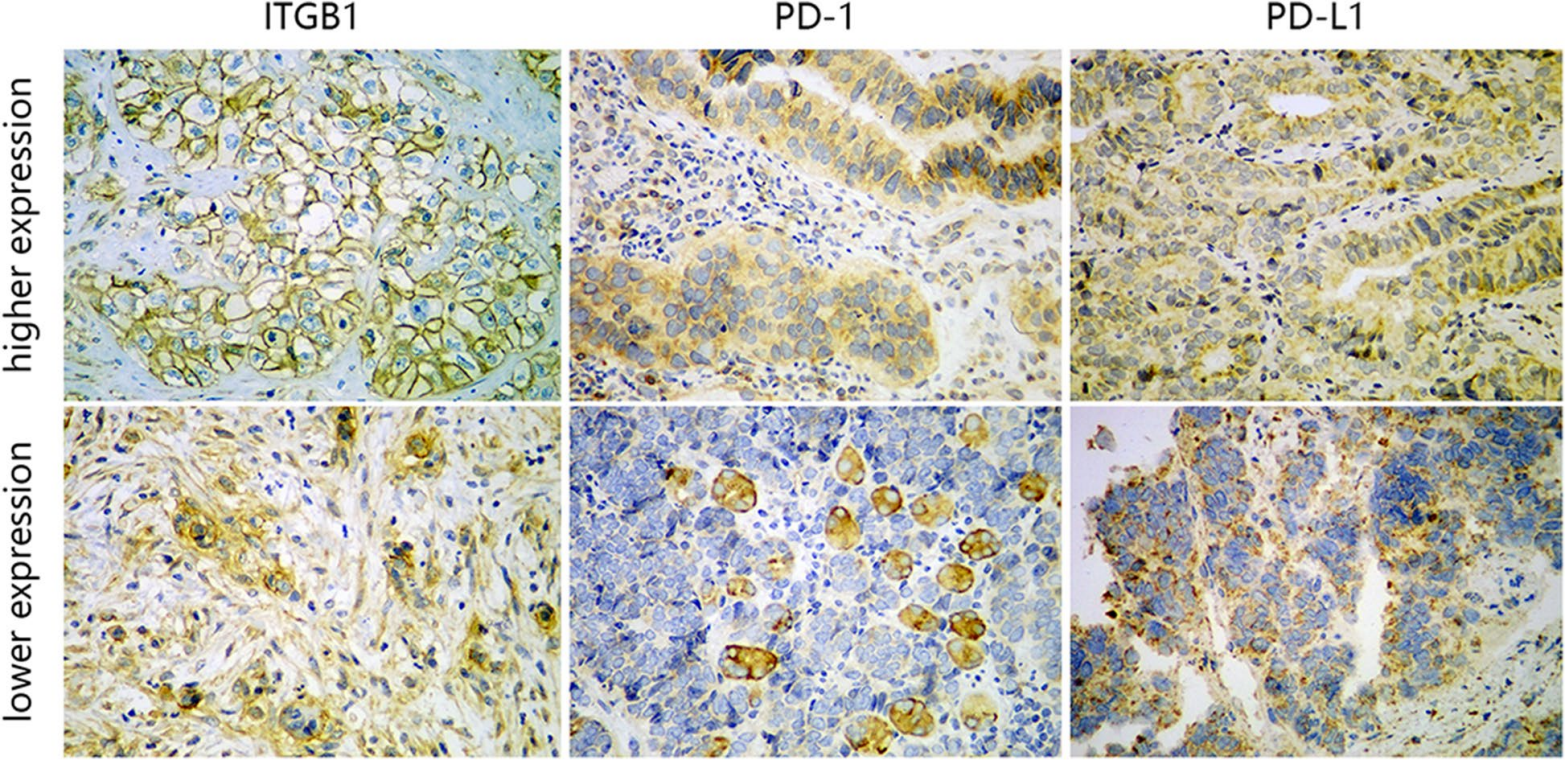
The immunohistochemistry images from wet lab showed up-regulated expression of ITGB1 in gastric tissues, and expression levels of PD-1 and PD-L1 were positively correlated with ITGB1

**Fig. 7:**
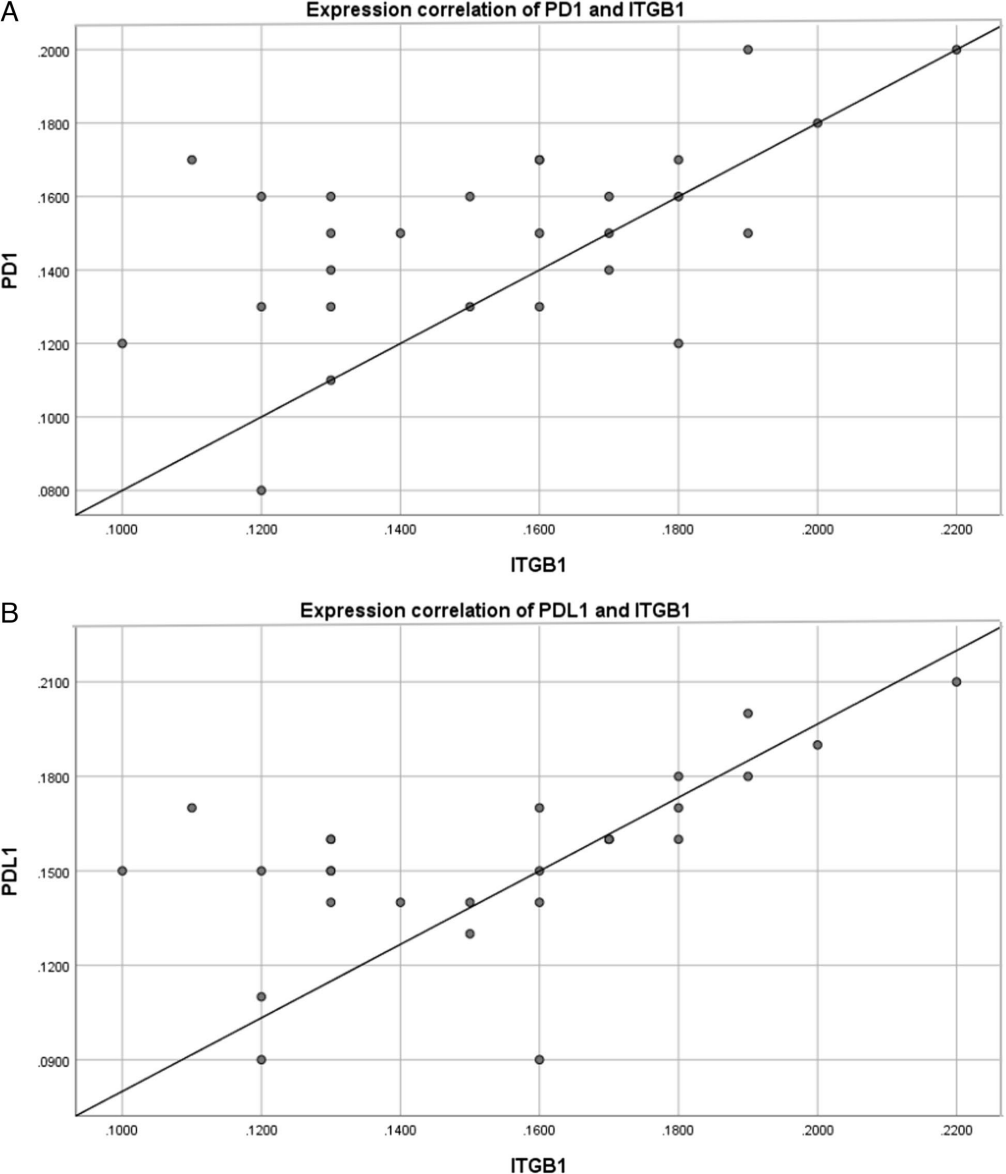
Correlation of indicators from clinical datas. **A** PD-1 expression is correlated with ITGB1 expression. **B** PD-L1 expression is correlated with ITGB1 expression

To further verify the expression of ITGB1 at the protein level and its correlation with PD-1 and PD-L1, we performed IHC on GC samples. Representative images representing positive expression are showed in Fig. 6. Similar to the results of the transcriptomic data, ITGB1 was up-regulated in gastric tissues. Correlation analysis indicated that levels of PD-1 and PD-L1 was positively correlated with ITGB1, with correlation coefficients 0.560 and 0.594, respectively (*P* < 0.01, Fig. 7A, B).

### ITGB1 could be used to predict the efficacy of PD-1 inhibitors

**Fig. 8:**
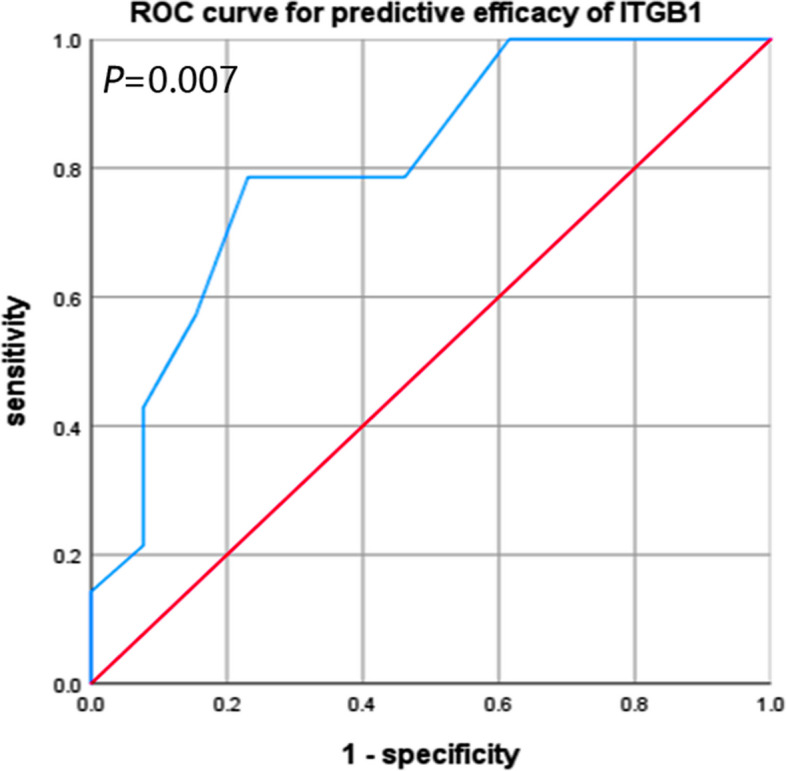
ROC curve indicate ITGB1 as an effective predictor for anti-PD-1 therapy

We collected clinical datas of 27 patients who had undergone immunotherapy with a PD-1 inhibitor. Patients were divided into two groups, effective with 14 cases, while non-effective with 13 cases. The two groups were comparable in terms of age, sex, tumor stage, ECOG (Eastern Cooperative Oncology Group) value, and adverse reactions (*P* > 0.05) (Table 3). To evaluate the ability of ITGB1 in predicting the efficacy of PD-1 inhibitor we performed ROC curve analysis. The area under the ROC curve was 0.808, which indicated that ITGB1 was an effective predictor for anti-PD-1 therapy (Fig. 8).

**Table 3:** Basic information of two groups

		Efficacy		*P* Value
		Effective	Non-Effective	
Sex	Male	10	10	0.851
	Female	4	3	
Age		65.21 ± 11.48	62.54 ± 7.81	0.345
ECOG Score	0	3	3	0.534
	1	11	10	
Side Effect	Grade 1	2	2	0.187
	Grade 2	12	11

### Expression of PD-L1 is correlated with ITGB1 in gastric cancer in vitro

**Fig. 9:**
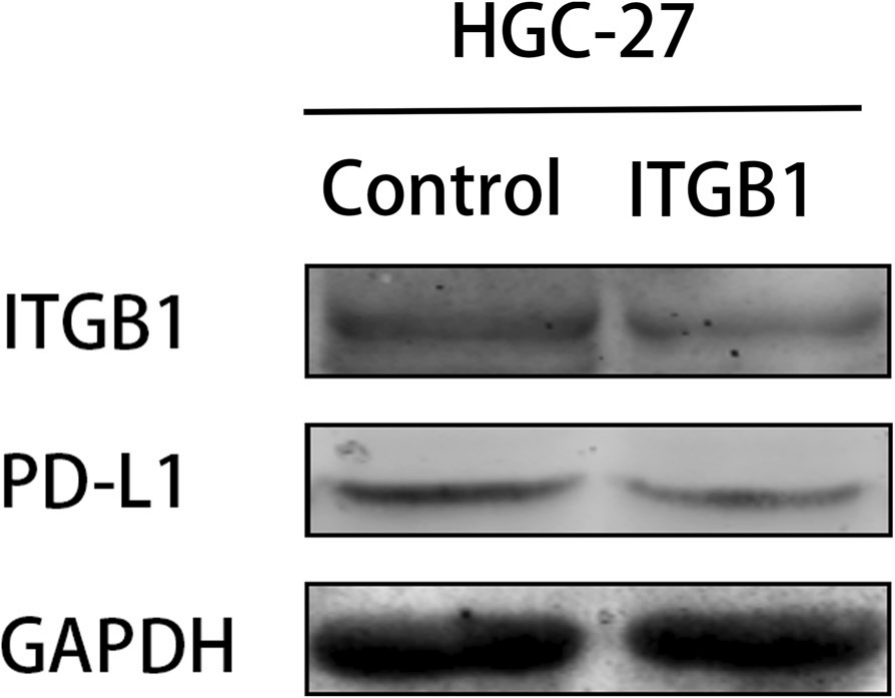
Result of western blotting indicate that expression of PD-L1 decreased after ITGB1 is knocked down

**Fig. 10:**
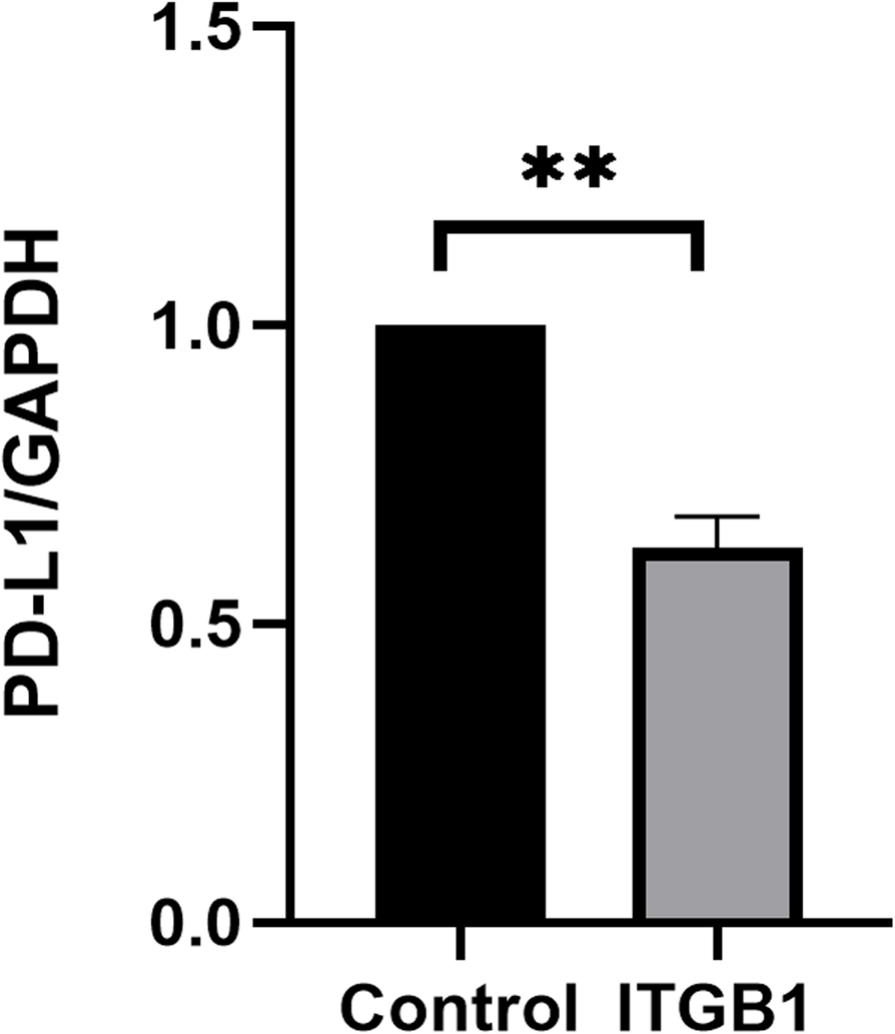
Expression of PD-L1 is declined statistically in observation group

To clarify the correlation of ITGB1 and PD-L1 in gastric cancer cells, ITGB1 was knocked down and PD-L1 expression was observed in gastric carcinoma cell line HGC-27. The results indicated that, expression of PD-L1 decreased by knock down of ITGB1, which suggest that ITGB1 may affect immunotherapy by promoting the expression of PD-L1 (**P < 0.01, Figs. 9 and 10).

## Disscussion

Immunotherapy had been approved in many kinds of advanced malignancies, such as lung cancer, melanoma, renal cancer, pancreatic cancer, colorectal cancer, as well as gastric cancer [27]. Despite the effectiveness of targeted therapy, the overall survival is still poor due to lack of efficacy predictors. Researchers found that, MX2 may be effective for sunitinib in therapy of renal carcinoma [28]. Stemness-related gene AC01097.3 is considered to be a novel potential therapeutic target for renal clear cell carcinoma and RNA-binding proteins for renal papillary cell carcinoma [29, 30]. lncRNAs were found to be signifcantly associated with the survival of patients with colon adenocarcinoma [31]. So far, there is no efficacy markers for immunotherapy of gastric cancer have been explored.

As mentioned above, immune infiltration may play an important role in the development of GC. Immune cells have been studied for biomarkers and evaluated for anti-PD1/PD-L1 efficacy, including tumor-infiltrating T cells (TILs) and macrophages [32]. Over-expression of EMT-related proteins has been observed in up to 63% of GC cases [33], while over-expression and amplification of EMT markers are significant prognostic indicators of poor survival outcomes in GC patients [34]. Several studies have confirmed some links between EMT and immune infiltration. SNAI1, an EMT-related gene, is associated with immune infiltration and can be used as a prognostic biomarker in gastrointestinal cancers [35]. Some studies also found that, tumor cells and immune cells could respond to similar stimuli by activating alike programs, leading to the occurrence of EMT and the infiltration of immunosuppressive cells. One of the most studied stimuli is TGF-β, which is a potent inducer of EMT [36]. Kang et al. found that [37], CDKN2A, CMTM8 and ILK which related with EMT and immune infiltration are promising prognostic biomarkers and may be potential therapeutic targets in colon cancer by participating in the TGF-β pathway. In addition, it inhibits the infiltration of immune cells and promotes the differentiation of suppressed or exhausted immune cells [38]. However, due to the complexity of human immune mechanisms, there are still many problems that need to be further addressed in GC immunotherapy, particularly in terms of immunotherapeutic biomarkers predicting and new effective therapeutic targets identifying.

Based on the studies above, we searched for genes associated with both immune infiltration and EMT as potentially effective markers for immunotherapy in GC. The integrin family is a class of heterodimeric trans-membrane mucins formed of two subunits in a 1:1 ratio, bound by non-covalent bonds. Integrins can remodel ECM structure, and play an important role in tumor formation and metastasis. In addition, over-expression of integrins lead to a decline in homogeneous adhesion and an increase in heterogeneous adhesion in GC. Within the integrin family, ITGB1 is the member most closely associated with GC metastasis [25]. ITGB1 is highly expressed in many malignant tumors, while studies have confirmed [39] correlations between the level of ITGB1 expression and the degree of differentiation, invasion, and lymphatic metastasis in GC. ITGB1 expression was shown to be significantly stronger in tumor tissue than in adjacent normal gastric mucosa, while stronger expression was also observed in poorly differentiated tumor tissue that had penetrated the serosa layer [40].

Previous studies have indicated the mechanisms of ITGB1 in gastric cancer progression. The Wnt/β-catenin signaling pathway has been identified to be a core mediator of signaling downstream of the oncogenic functions of integrin family members. ITGB1 is one of the upstream molecules of the Wnt/β-catenin signaling pathway and is correlated with tumor immune suppression. Researchers found that, expression of β-catenin in nucleus and cytoplasm was upregulated in ITGB1-positive group compared with the negative group [41]. Served as fundamental component in extracellular matrix, Type I collagen could upregulate the expression of BCL9L through ITGB1, resulting in the activation β-catenin signaling pathway, thereby contributing to the gastric cancer development [42]. At the same time, as a cytokine receptor, integrins and their kinases were also necessary for the activation of TGF-β and its induction of cellular EMT. During EMT in cells, TGF-β can enhanced the expression of integrins and made them more exposed on the cell membrane surface, thereby regulating cell–matrix interactions [43].

Our study indicated that ITGB1 is a carcinogenic factor expressed more highly in tumors with poor prognosis. High levels of ITGB1 resulted in reduced OS rates, worse pathology G-staging and tumor T-staging which is indicative of clinical progression and consistent with previous reports. Previous studies have reported that high density of TIL and increased numbers of CD3 + or CD8 + T cells are associated with favorable prognoses in GC patients [44, 45]. Based on the status of TIL distribution in tumors or peritumoral stroma, several studies have suggested the concept of different immune phenotypes: inflamed (TILs located intratumorally), excluded (TILs retained in the peritumoral stroma), and immune deserts (sparse TILs in both tumor nests and stroma) [46–48]. Tumors with an inflamed phenotype showed increased PD-L1 expression and immune cell numbers and better response to ICI treatment [47, 48]. Recently, the study of Kim et al. showed a significant association between TIL numbers and OS in GC [48]. In our study, we found that expression of ITGB1 was positively correlated with CD8 + T cells, macrophages, and dendritic cells, while negatively correlated with B cells; there was no significant association with CD4 + T cells. These findings indicate that GC samples with ITGB1 overexpression also showed elevated immune cell infiltration. This was also partially consistent with the study of Wang et al. [49], which demonstrated activation of CD4 + memory T cells and high levels of infiltration of monocytes, M0 macrophages, M0 macrophages, and M2 macrophages in gastric tumor samples. These results may explain why better clinical efficacy is achieved in patients with higher ITGB1 expression.

In the phase II KEYNOTE-59 trial [50], PD-L1 + patients with advanced or metastatic GC showed durable responses to pembrolizumab treatment. The recent phase III CheckMate 649 study [51] also showed that nivolumab plus chemotherapy demonstrated statistically significant improvements in both median OS and progression-free survival (PFS) in patients with a PD-L1 CPS ≥ 5 (OS, hazard ratio [HR] 0.71, 98.4% confidence interval [CI] 0.59–0.86, *P* < 0.0001; and PFS, HR 0.68, 98% CI 0.56–0.81, *P* < 0.0001). Additionally, patients with a PD-L1 CPS ≥ 1 showed a significant increase in OS (HR 0.77, 99.3% CI 0.6–0.92; *P* = 0.0001). Based on these results, the Food and Drug Administration has approved the use of PD-L1 inhibitors for anti-tumor therapy.

Bioinfamatics studies found that [52], EMT-immune-related genes (EIRG) score can be used as a biomarker to identify and screen patients for immunotherapy. And patients with lower EIRG_score responded better to immunotherapy. However, there is no relevant study on whether ITGB1 is suitable for an indicator of immunotherapy. In this study, we found that ITGB1 was positively correlated with the expression of PD-L1. This was confirmed by correlation analysis of the clinical data, which produced correlation coefficients of 0.594. Meanwhile, in vitro cell experiment further verified this point. ITGB1 expression was also analyzed in the effective and non-effective PD-1 treatment groups. The area under the ROC curve was 0.808, which indicated ITGB1 as an effective predictor for anti-PD-1 therapy. And it is the first time that ITGB1 considered as an efficacious indicator for immunotherapy.

## Conclusion

This study found that higher expression of ITGB1 was correlated with poor prognosis of GC and ITGB1 may promote GC by regulation of immune checkpoints. PD-L1 expression was positively correlated with ITGB1, which indicate that ITGB1 may be an effective predictor for GC immunotherapy.

### Supplementary Information

**Additional file 1.**

## Data Availability

The datasets GSE118916 and GSE79973 are available in the GEO database (http://www.ncbi.nlm.nih.gov/geo). Meanwhile, the TCGA repository (https://portal.gdc.cancer.gov/projects/TCGA) was utilized, under the accession code: Stomach adenocarcinoma (STAD). GTEx data were used by GEPIA online database.

## Abbreviations

GC: Gastric cancer
EMT: Epithelial-mesenchymal transition
TCGA: The Cancer Genome Atlas
DEGs: Different expression genes
RNA-seq: RNA sequencing
IHC: Immunohistochemistry
ROC curves: Receiver operating characteristic curves

## References

[1] Sung H, Ferlay J, Siegel RL, Laversanne M, Soerjomataram I, Jemal A, Bray F (2021). Global cancer statistics 2020: GLOBOCAN estimates of incidence and mortality worldwide for 36 cancers in 185 countries. CA Cancer J Clin.

[2] Yamashita K, Hosoda K, Niihara M, Hiki N (2021). History and emerging trends in chemotherapy for gastric cancer. Ann Gastroenterol Surg.

[3] Koizumi W, Narahara H, Hara T, Takagane A, Akiya T, Takagi M, Miyashita K, Nishizaki T, Kobayashi O, Takiyama W, Toh Y, Nagaie T, Takagi S, Yamamura Y, Yanaoka K, Orita H, Takeuchi M (2008). S-1 plus cisplatin versus S-1 alone for first-line treatment of advanced gastric cancer (SPIRITS trial): a phase III trial. Lancet Oncol.

[4] Chung HC, Bang YJ, Fuchs CS, Qin SK, Satoh T, Shitara K, Tabernero J, Cutsem EV, Alsina M, Cao ZA, Lu J, Bhagia P, Shih CS, Janjigian YY (2021). First-line pembrolizumab/placebo plus trastuzumab and chemotherapy in HER2-positive advanced gastric cancer: KEYNOTE-811. Future Oncol.

[5] Wilke H, Muro K, Cutsem EV, Oh SC, Bodoky G, Shimada Y, Hironaka S, Sugimoto N, Lipatov O, Kim TY, Cunningham D, Rougier P, Komatsu Y, Ajani J, Emig M, Carlesi R, Ferry D, Chandrawansa K, Schwartz JD, Ohtsu A, RAINBOW Study Group (2014). Ramucirumab plus paclitaxel versus placebo plus paclitaxel in patients with previously treated advanced gastric or gastro-oesophageal junction adenocarcinoma (RAINBOW): a doubleblind, randomised phase 3 trial. Lancet Oncol.

[6] Li KX, Zhang A, Li XY, Zhang HT, Zhao LM (2021). Advances in clinical immunotherapy for gastric cancer. Biochim Biophys Acta Rev Cancer.

[7] Robert C, Long GV, Brady B, Dutriaux C, Maio M, Mortier L, Hassel JC, Rutkowski P, McNeil C, Kalinka-Warzocha E, Savage KJ, Hernberg MM, Lebbé C, Charles J, Mihalcioiu C, Chiarion-Sileni V, Mauch C, Cognetti F, Arance A, Schmidt H, Schadendorf D, Gogas H, Lundgren-Eriksson L, Horak C, Sharkey B, Waxman IM, Atkinson V, Ascierto PA (2015). Nivolumab in previously untreated melanoma without BRAF mutation. N Engl J Med.

[8] Reck M, Abreu DR, Robinson AG, Hui R, Csőszi T, Fülöp A, Gottfried M, Peled N, Tafreshi A, Cuffe S, O'Brien M, Rao S, Hotta K, Leiby MA, Lubiniecki GM, Yue S, Rangwala R, Brahmer JR, KEYNOTE-024 Investigators (2016). Pembrolizumab versus chemotherapy for PD-L1-positive non-small-cell lung cancer. N Engl J Med.

[9] Herbst RS, Baas P, Kim DW, Felip E, Gracia JLP, Han JY, Molina J, Kim JH, Arvis CD, Ahn MJ, Majem M, Fidler MJ, de Castro G, Garrido M, Lubiniecki GM, Yue S, Ellie IM, Marisa DF, Garon EB (2016). Pembrolizumab versus docetaxel for previously treated, PD-L1-positive, advanced non-small-cell lung cancer (KEYNOTE-010): a randomised controlled trial. Lancet.

[10] Motzer RJ, Escudier B, McDermott DF, George S, Hammers HJ, Srinivas S, Tykodi SS, Sosman JA, Procopio G, Plimack ER, Castellano D, Choueiri TK, Gurney H, Donskov F, Bono P, Wagstaff J, Gauler TC, Ueda T, Tomita Y, Schutz FA, Kollmannsberger C, Larkin J, Ravaud A, Simon JS, Xu LA, Waxman IM, Sharma P, CheckMate 025 Investigators (2015). Nivolumab versus everolimus in advanced renal-cell carcinoma. N Engl J Med.

[11] Pardoll DM (2012). The blockade of immune checkpoints in cancer immunotherapy. Nat Rev Cancer.

[12] Kawazoe A (2021). Current status of immunotherapy for advanced gastric cancer. Japanese J Clin Oncol.

[13] Sharpe AH, Pauken KE (2018). The diverse functions of the PD1 inhibitory pathway. Nat Rev Immunol.

[14] Song JQ, Wei RY, Huo SY, Gao JP, Liu XW (2022). Metastasis related epithelial-mesenchymal transition signature predicts prognosis and response to immunotherapy in gastric cancer. Front Immunol.

[15] Wu C, Zhuang Y, Jiang S, Liu SL, Zhou JY, Wu J, Teng YH, Xia BM, Wang RP, Zou X (2016). Interaction between Wnt/beta-catenin pathway and microRNAs regulates epithelial-mesenchymal transition in gastric cancer (Review). Int J Oncol.

[16] Cantelli G, Crosas-Molist E, Georgouli M, Sanz-Moreno V (2017). TGFβ-in-duced transcription in cancer. Semin Cancer Biol.

[17] Zheng HX, Cai YD, Wang YD, Cui XB, Xie TT, Li WJ, Peng L, Zhang Y, Wang ZQ, Wang J, Jiang B (2013). Fas signaling promotes motility and metastasis through epithelial-mesenchymal transition in gastrointestinal cancer. Oncogene.

[18] Zheng HX, Li WJ, Wang YD, Liu ZZ, Cai YD, Xie TT, Shi M, Wang ZQ, Jiang B (2013). Glycogen synthase kinase3 beta regulates Snail and beta-catenin expression during Fas-induced epithelial-mesenchymal transition in gastrointestinal cancer. Eur J Cancer.

[19] Lamouille S, Xu J, Derynck R (2014). Molecular mechanisms of epithelial-mesechymal transition. Nat Rev Mol Cell Biol.

[20] Jiang YY, Zhan HX (2020). Communication between EMT and PD-L1 signaling: new insights into tumor immune evasion. Cancer Lett.

[21] Qiu BQ, Lin XH, Lai SQ, Lu F, Lin K, Long X, Zhu SQ, Zou HX, Xu JJ, Liu JC, Wu YB (2021). ITGB1-DT/ARNTL2 axis may be a novel biomarker in lung adenocarcinoma: a bioinformatics analysis and experimental validation. Cancer Cell Int.

[22] Wang X, Li TZ (2021). Ropivacaine inhibits the proliferation and migration of colorectal cancer cells through ITGB1. Bioengineered.

[23] Zhuang HK, Zhou ZX, Ma ZY, Li ZC, Liu CS, Huang SZ, Zhang CZ, Hou BH (2020). Characterization of the prognostic and oncologic values of ITGB superfamily members in pancreatic cancer. J Cell Mol Med.

[24] Li YX, Sun C, Tan YG, Zhang HY, Li YC, Zou HW (2021). ITGB1 enhances the radioresistance of human non-small cell lung cancer cells by modulating the DNA damage response and YAP1-induced epithelial-mesenchymal transition. Int J Biol Sci.

[25] Lv YL, Shan YJ, Song LN, Zhao YF, Lai RX, Su JY, Zhang XY (2021). Type I collagen promotes tumor progression of integrin β1 positive gastric cancer through a BCL9L/β-catenin signaling pathway. Aging.

[26] Wu TT, Tang CJ, Tao RC, Yong XZ, Jiang QZ, Feng C (2021). PD-L1-mediated immunosuppression in oral squamous cell carcinoma: relationship with macrophage infiltration and epithelial to mesenchymal transition markers. Front Immuno.

[27] Wang D, Wu X, Sun Y (2022). Therapeutic targets and biomarkers of tumor immunotherapy: response versus non-response. Signal Transduct Target Ther.

[28] Wei Y, Chen X, Ren X, Wang B, Zhang Q, Bu H, Qian J, Shao P (2021). Identification of MX2 as a novel prognostic biomarker for sunitinib resistance in clear cell renal cell carcinoma. Front Genet.

[29] Jiang SL, Ren XH, Liu SY, Lu ZW, Xu AM, Qin C, Wang ZJ (2021). Integrated analysis of the prognosis-associated rna-binding protein genes and candidate drugs in renal papillary cell carcinoma. Front Genet.

[30] Liu YQ, Wang JW, Li L, Qin HB, Wei Y, Zhang X, Ren XH, Ding W, Shen XD, Li GY, Lu ZW, Zhang D, Qin C, Tao LS, Chen XL (2022). AC010973.2 promotes cell proliferation and is one of six stemness-related genes that predict overall survival of renal clear cell carcinoma. Sci Rep.

[31] Wu DJ, Yin ZH, Ji YS, Li L, Li YX, Meng FQ, Ren XH, Xu M (2021). Identifcation of novel autophagy-related lncRNAs associated with a poor prognosis of colon adenocarcinoma through bioinformatics analysis. Sci Rep.

[32] Thompson JC, Hwang WT, Davis C, Deshpande C, Jeffries S, Rajpurohit Y, Krishna V, Smirnov D, Verona R, Lorenzi MV, Langer CJ, Albelda SM (2020). Gene signatures of tumor inflammation and Epithelial-to-Mesenchymal Transition (EMT) predict responses to immune checkpoint blockade in lung cancer with high accuracy. Lung Cancer.

[33] Choi S, Park S, Kim H, Kang SY, Ahn S, Kim KM (2022). Gastric cancer: mechanisms, biomarkers, and therapeutic approaches. Biomedicines.

[34] Sohn SH, Kim B, Sul HJ, Kim YJ, Kim HS, Kim HT, Seo JB, Koh Y, Zang DY (2019). INC280 inhibits Wnt/β-catenin and EMT signaling pathways and its induce apoptosis in diffuse gastric cancer positive for c-MET amplification. BMC Res Notes.

[35] Fang J, Ding Z (2020). SNAI1 is a prognostic biomarker and correlated with immune infiltrates in gastrointestinal cancers. Aging (Albany NY).

[36] David CJ, Massagué J (2018). Contextual determinants of TGFβ action in development, immunity and cancer. Nat Rev Mol Cell Bio.

[37] Kang N, Xie X, Zhou X, Wang Y, Chen S, Qi R, Liu T, Jiang H (2022). Identification and validation of EMT-immune-related prognostic biomarkers CDKN2A, CMTM8 and ILK in colon cancer. BMC Gastroenterol.

[38] Johansson J, Tabor V, Wikell A, Jalkanen S, Fuxe J (2015). TGF-β1-induced Epithelial-mesenchymal transition promotes monocyte/macrophage properties in breast cancer cells. Front Oncol.

[39] Lee JS, Won HS, Sun DS, Hong JH, Ko YH (2018). Prognostic role of tumor-infiltrating lymphocytes in gastric cancer: A systematic review and meta-analysis. Medicine (Baltimore).

[40] Figueiredo J, Ferreira RM, Xu H, Gonçalves M, Carvalho AB, Cravo J, Maia AF, Carneiro P, Figueiredo C, Smith ML, Stamenović D, Morais-de-Sá E, Seruca R (2022). Integrin β1 orchestrates the abnormal cell-matrix attachment and invasive behaviour of E-cadherin dysfunctional cells. Gastric Cancer.

[41] Gu W, Sun H, Zhang M, Mo S, Tan C, Ni S, Yang Z, Wang Y, Sheng W, Wang L (2023). ITGB1 as a prognostic biomarker correlated with immune suppression in gastric cancer. Cancer Med.

[42] Lv Y, Shan Y, Song L, Zhao Y, Lai R, Su J, Zhang X (2021). Type I collagen promotes tumor progression of integrin β1 positive gastric cancer through a BCL9L/β-catenin signaling pathway. Aging (Albany NY).

[43] Annes JP, Chen Y, Munger JS, Rifkin DB (2004). Integrin alphaVbeta6-mediated activation of latent TGF-beta requires the latent TGF-beta binding protein-1. J Cell Biol.

[44] Zhang D, He W, Wu C, Tan Y, He Y, Xu B, Chen L, Li Q, Jiang J (2019). Scoring system for tumor-infiltrating lymphocytes and its prognostic value for gastric cancer. Front Immunol.

[45] Hegde PS, Karanikas V, Evers S (2016). The where, the when, and the how of immune monitoring for cancer immunotherapies in the era of checkpoint inhibition. Clin Cancer Res.

[46] Chen DS, Mellman I (2017). Elements of cancer immunity and the cancer-immune set point. Nature.

[47] Mariathasan S, Turley SJ, Nickles D, Castiglioni A, Yuen K, Wang YL, Kadel EE, Koeppen H, Astarita JL, Cubas R, Jhunjhunwala S, Banchereau R, Yang Y, Guan YH, Chalouni C, Ziai J, Şenbabaoğlu Y, Santoro S, Sheinson D, Hung J, Giltnane JM, Pierce AA, Mesh K, Lianoglou S, Riegler J, Carano R, Eriksson P, Höglund M, Somarriba L, Halligan DL, Heijden M, Loriot Y, Rosenberg JE, Fong L, Mellman I, Chen DS, Green M, Derleth C, Fine G, Hegde PS, Bourgon R, Powles T (2018). TGFβ attenuates tumour response to PD-L1 blockade by contributing to exclusion of T cells. Nature.

[48] Kim H, Heo YJ, Cho YA, Kang SY, Ahn S, Kim KM (2022). Tumor immune microenvironment is influenced by frameshift mutations and tumor mutational burden in gastric cancer. Clin Transl Oncol.

[49] Wang H, Rong J, Zhao Q, Song C, Zhao R, Chen S, Xie Y. Identification and Validation of Immune Cells and Hub Genes in Gastric Cancer Microenvironment. Dis Markers. 2022;2022:8639323. https://www.hindawi.com/journals/dm/2022/8639323/, https://pubmed.ncbi.nlm.nih.gov/35422890/. 10.1155/2022/8639323 PMC9005323 35422890

[50] Fuchs CS, Doi T, Jang RW, Muro K, Satoh T, Machado M, Sun WJ, Jalal SI, Shah MA, Metges JP, Garrido M, Golan T, Mandala M, Wainberg ZA, Catenacci DV, Ohtsu A, Shitara K, Geva R, Bleeker J, Ko AH, Ku G, Philip P, Enzinger PC, Bang YJ, Levitan D, Wang JD, Rosales M, Dalal RP, Yoon HH (2018). Safety and effificacy of pembrolizumab monotherapy in patients with previously treated advanced gastric and gastroesophageal junction cancer: phase 2 clinical KEYNOTE-059 Trial. JAMA Oncol.

[51] Janjigian YY, Shitara K, Moehler M, Garrido M, Salman P, Shen L, Wyrwicz L, Yamaguchi K, Skoczylas T, Bragagnoli AC, Liu TS, Schenker M, Yanez P, Tehfe M, Kowalyszyn R, Karamouzis MV, Bruges R, Zander T, Cid RP, Hitre E, Feeney K, Cleary JM, Poulart V, Cullen D, Lei M, Xiao H, Kondo K, Li MS, Ajani JA (2021). First-line nivolumab plus chemotherapy versus chemotherapy alone for advanced gastric, gastrooesophageal junction, and oesophageal adenocarcinoma (CheckMate 649): a randomised, open-label, phase 3 trial. Lancet.

[52] Zhang X, Li Y, Hu P, Xu L, Qiu H (2022). Identification of molecular patterns and prognostic models of epithelial-mesenchymal transition and immune-combined index in the gastric cancer. Front Pharmacol.

