# Supplementary figures and images for: Evaluation of ITGB1 expression as a predictor of the therapeutic effects of immune checkpoint inhibitors in gastric cancer
Source: BMC Gastroenterol. 2023 Sep 4;23:298. doi: 10.1186/s12876-023-02930-0 (PMC10478479; doi:10.1186/s12876-023-02930-0)

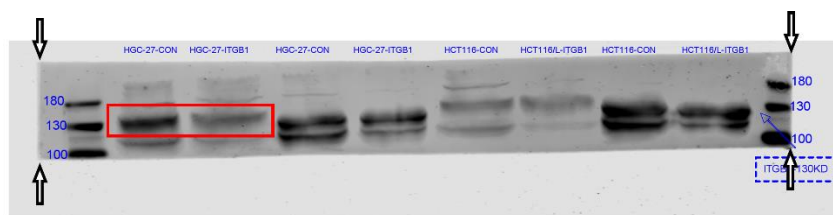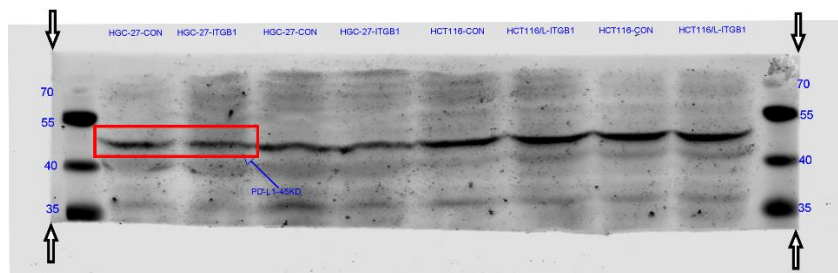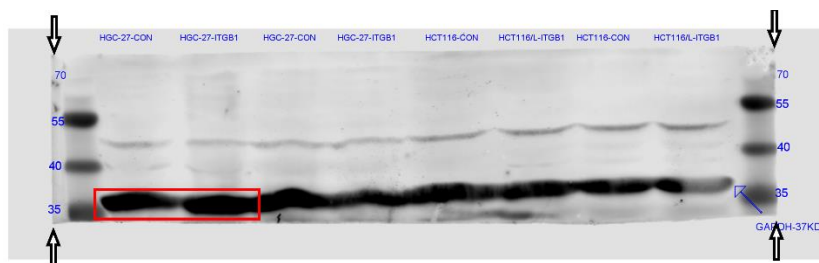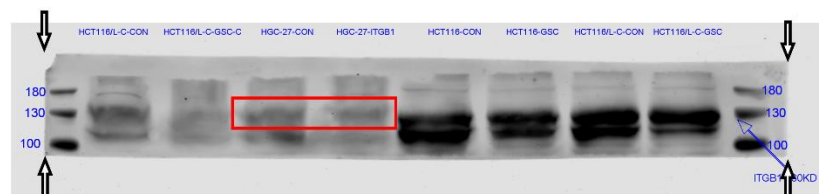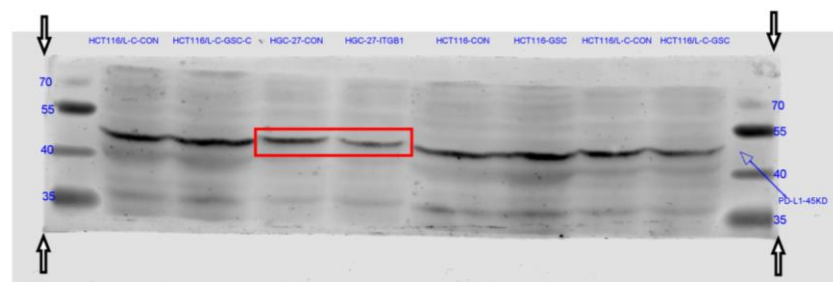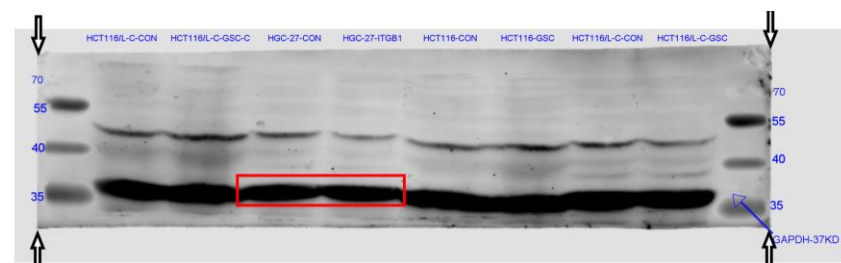

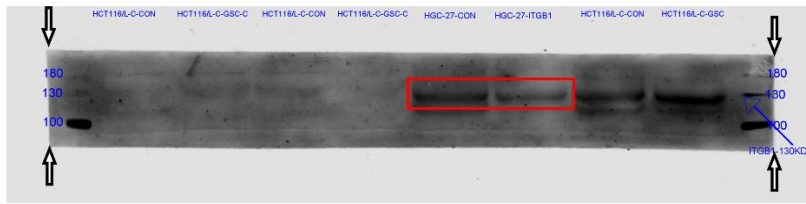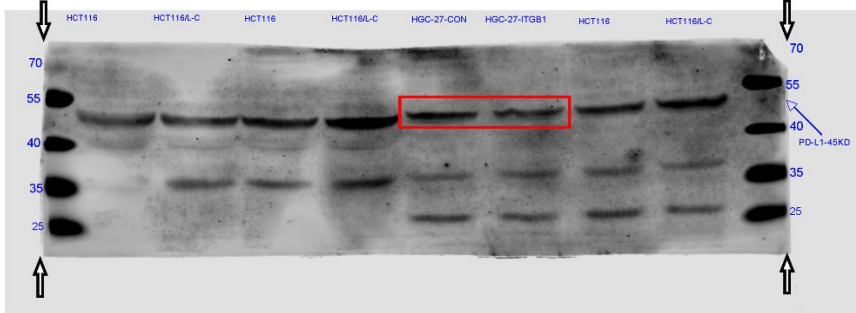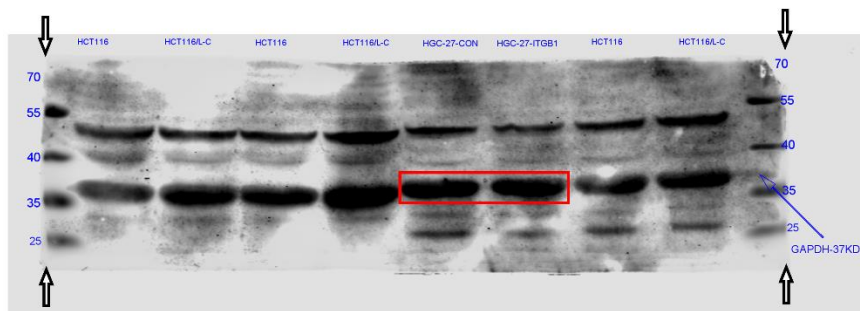

Supplement: Supplementary file 1 — Additional file 1. [file 12876_2023_2930_MOESM1_ESM.pdf]
